# Nonfunctional Pancreatic Neuroendocrine Carcinoma With Isolated Retroperitoneal Metastasis: A Case Report and Literature Review

**DOI:** 10.1097/MPA.0000000000001468

**Published:** 2020-04-10

**Authors:** Haoxiang Zhang, Dong Shang

**Affiliations:** Department of General Surgery, The First Affiliated Hospital of Dalian Medical University, Dalian, China shangdong@dmu.edu.cn

To the Editor:

Pancreatic neuroendocrine tumors (pNETs) are rare neuroendocrine neoplasms, with an estimated incidence of approximately 1 per million per year, about 3% of primary pancreatic tumors.^1^ Most of them are nonfunctional tumors (50%–75%).^2^ Newly published 2010 World Health Organization (WHO) classification classified them into three distinct types: neuroendocrine tumor grade 1 (G1), neuroendocrine tumor grade 2 (G2), and neuroendocrine carcinoma (G3).^3^ Pancreatic neuroendocrine carcinoma (pNEC) only amounts to about 2% to 3% of all pNETs. Here we demonstrated a rare case of pNEC with isolated retroperitoneal metastasis. It served as the only reported case of pNEC metastases spread to the retroperitoneal preceded to other organs.

## CASE REPORT

**FIGURE 1:**
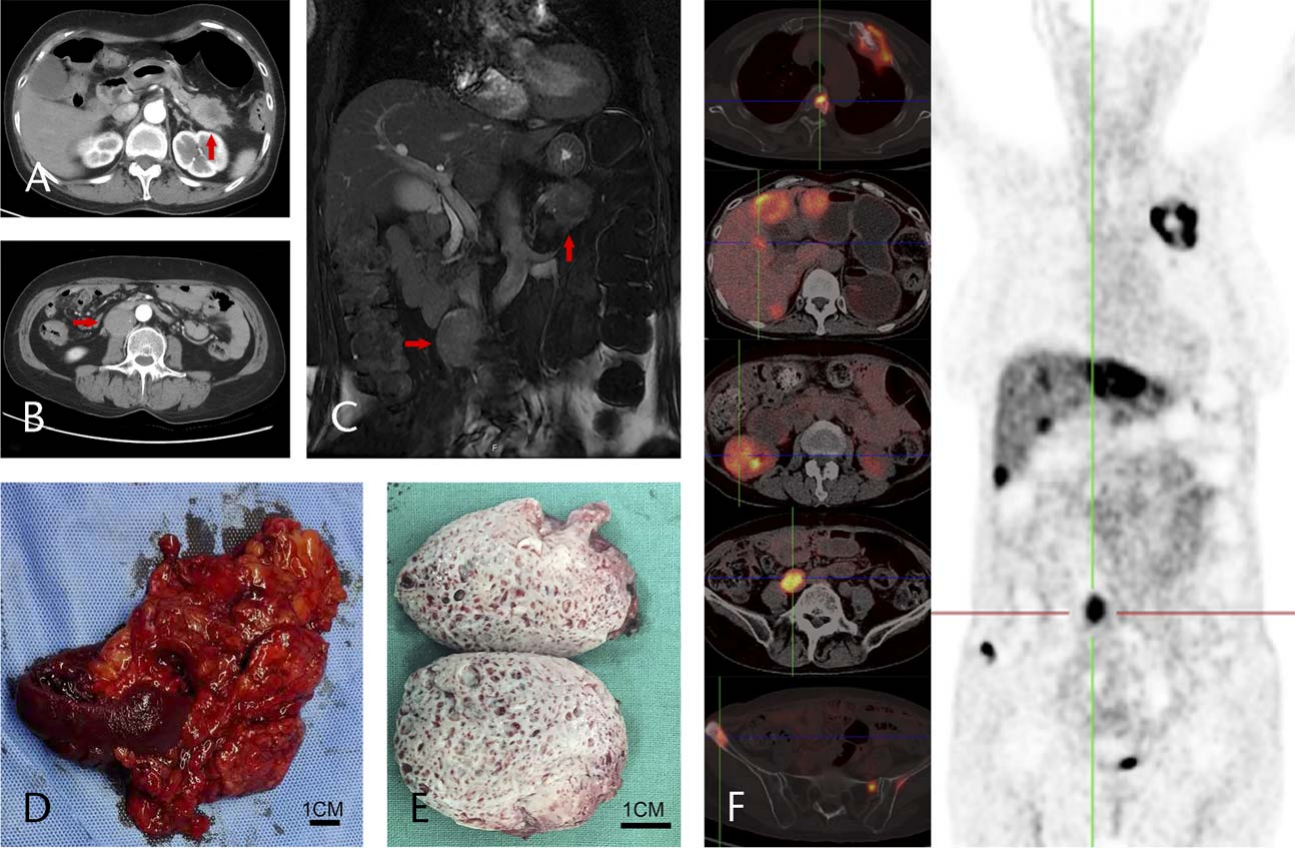
A, Pre-operative arterial phase CT image reveals a 2.82 × 3.71 cm poorly enhancing mass is defined in the pancreatic tail portion (↑). B, A 3.09-cm poorly enhanced nodule is seen in the right retroperitoneal, in front of right lumbar muscle (→). C, Pancreatic tail mass (↑) and retroperitoneal nodule (→) showed in preoperative magnetic resonance imaging. Resected specimens: (D) Pancreatic tail mass and (E) cutout view of the retroperitoneal nodule. F, ^18^F-Octreotide PET/CT showing increased tracer uptake in bones, liver, right kidney, and retroperitoneal. Hardly any uptake of ^18^F-FDG is evident at the same sites.

A 61-year-old female was referred to our hospital with gallbladder polyps and right upper quadrant pain. We discovered a 3.55-cm mass in the tail of the pancreas, a 3.92-cm mass in the right retroperitoneal, and right retroperitoneal lymph node enlargement (Figs. 1A-C). Tumor markers showed that carcinoembryonic antigen was 17.48 ng/mL (reference range, 0–5 ng/mL) and alpha-fetoprotein was 7.99 IU/mL (reference range, 0–5.8 IU/mL). The patient underwent distal pancreatectomy + splenectomy + regional lymph nodes dissection + retroperitoneal mass resection + cholecystectomy in our hospital. Upon microscopic analysis, the pancreatic tail was nodule confirmed as pNEC, and the retroperitoneal mass was distant metastasis (Figs. 1D-E). The mitotic count was 5 to 8 per 10 high-power field. Immunohistochemical staining revealed CD117, (+); CD56(NK-1), (+); CK19, (partially +); CK7, (−); CgA, (+); Ki-67, (20%–30%); Syn, (+). There were no lymph node metastases. The pathological diagnosis was T2, N0, M1, stage IV, with an R0 resection.

After 20 months of regular follow-up, some liver, bone, and right kidney lesions with retroperitoneal metastasis recurrence were found by abdominal computed tomography (CT). Further fluorine-18-fluorodeoxyglucose (^18^F-FDG) and fluorine-18-octreotide (^18^F-Octreotide) positron emission tomography (PET)/CT showed abnormal uptake of ^18^F-Octreotide was observed in newly found lesions and retroperitoneal metastasis recurrent, which were considered as NEC metastasis tumors (Fig. 1F). Hardly any uptake of ^18^F-FDG is evident at the same sites. The patient underwent further medical treatment (everolimus, 10 mg/d) in oncology. Unfortunately, the effect of medical treatment was minimal and the patient ultimately succumbed 25 months after the surgery.

## DISCUSSION

Pancreatic neuroendocrine tumors are extremely rare, and the biological behavior of pNETs is unpredictable. Neuroendocrine carcinoma is defined as carcinomas that have been reckoned as poorly differentiated tumors with highly invasive nature and high proclivity for metastatic dissemination. Making early diagnosis and treatment seems complicated if they are nonfunctional and thus have no special symptoms or signs at an early stage. Most of them are incidentally detected on imaging and the patients already have distant metastases, as in this case. Computed tomography and magnetic resonance imaging can be used to establish the location of the primary NETs and help guide the proper surgical or medical treatment. Recent various meta-analyses or series show that (68)Ga-labeled somatostatin analogs PET/CT have higher sensitivity (92%), higher specificity (88%), higher accuracy (93%), and empowers whole-body assessment of disease extent.^4^ The functional imaging of NETs can be also used to evaluate malignancy because different grades of NETs exhibit different receptor expression or metabolic pathways.^5^

The microscopic analysis combined with immunohistochemistry is the “gold standard” for the diagnosis of pNETs. Chromogranin A (CgA) and synaptophysin (Syn) are neuroendocrine markers.^6^ Histopathological classification of pNETs can be categorized based on the mitotic count and the Ki-67 proliferation index according to 2010 WHO classification of gastropancreatic-neuroendocrine neoplasms. On this measure, the high of two is adopted for categorization, our case can be classified as G3. Some authors speculate that NEC with a Ki-67 index below 55%, can be called a new category, had better survival and lower relative risk.^7^ Many scholars hold the view that NEC may not have only 1 entity. By analyzing 2158 cases from the Surveillance, Epidemiology, and End Results database, grading and systemic metastases are considered to have a significant impact on survival.^8^

Surgical resection with regional lymph node dissection is the only cure for patients with pancreatic NETs, but the role of chemotherapy after curative resection is still unclear. The National Comprehensive Cancer Network guideline recommends if complete resection is possible, resect the primary tumor and metastases with a regular postoperative review is enough.^9^ In contrast, for poorly differentiated (high-grade) extrapulmonary NECs, the North American Neuroendocrine Tumor Society guideline believes that it is unlikely to be curative by surgery alone. Chemotherapy (4 to 6 cycles of carboplatin and etoposide or cisplatin) after surgery is recommended.^10^

In summary, owing to the nonfunction, most of the pNETs can only be incidentally discovered by imaging. Surgical resection remains the only cure. Positron emission tomography/CT is one of best imaging techniques for whole body assessment of disease extent and valuable surgical or medical treatment. Chemotherapy after curative resection of pNEC may influence their prognosis. Medical management for advanced pNETs is of equal importance.

**Haoxiang Zhang, MD** **Dong Shang, MD, PhD**
Department of General Surgery
The First Affiliated Hospital
of Dalian Medical University
Dalian, China
shangdong@dmu.edu.cn
